# Primary Health Center: Can it be made mobile for efficient healthcare services for hard to reach population? A state-of-the-art review

**DOI:** 10.1007/s44250-023-00017-x

**Published:** 2023-01-23

**Authors:** Md Haseen Akhtar, Janakarajan Ramkumar

**Affiliations:** Indian Institute of Technology, Kanpur, India

**Keywords:** Primary Health Center (PHC), Mobile Medical Unit (MMU), Mobile Health Unit (MHU), Hard-to-reach populations, Primary healthcare services, Healthcare delivery model, Mobile Primary Health Center (mPHC)

## Abstract

Indian healthcare system is in immediate need of a new healthcare delivery model to increase healthcare accessibility and improve the health outcomes of the marginalized. Inaccessibility and underutilization of Primary Health Centers (PHCs) disproportionately affect people living in remote areas. It is thus imperative for the designers, engineers, health professionals, and policymakers to come together with a collaborative mindset to develop innovative interventions that sustainably manage the accessibility of PHCs at large, promote preventive health, and thus improve the health outcomes of hard-to-reach communities. This article examines the available literature on barriers to primary healthcare in Indian context, the reason of failure of PHCs and the way forward. The article further analysis literature on existing Mobile Medical Units (MMUs) as an alternate solution to conventional PHCs and attempt to extract the major lessons to propose a mobile Primary Health Center (mPHC) in contrast to the existing conventional static PHCs. The intention is to find out the research gaps in the existing literature and try to address the same for future researchers, designers, engineers, health professionals and policy makers to think forward to make this idea of a mobile Primary Health Center (mPHC), as the main delivery model to cater basic healthcare services to the underserved communities.

## Highlights

- It is evident from the study that Indian (or any third world countries) healthcare system needs a new delivery model, arguably, a mobile Primary Health Center (mPHC).
- This study revealed the potential of Primary Health Center to be mobile and cater to wide range of services to the remotest of the population.
- State of the art facilitated the development of a more efficient mobile healthcare system in future.
- Need of collaborative intervention on the part of stakeholders on the adoption and operationalization of a mobile PHC.

## Introduction

The Indian healthcare scenario presents a wide range of healthcare adversities. At one end are the high glazed buildings with state-of-the-art facilities affordable only to the upper class living in urban India. At the other end are the norms for many trying to get the basic healthcare services by travelling miles and spending the complete depth and breadth of their pocket but still not satisfied with the quality of services. Thus, it is imperative to ask the following questions: How can the socially disadvantaged, economically challenged and the systematically marginalized should be included as “everyone” for providing basic healthcare services? How the reachability of providing healthcare services should not be limited to plain areas but should also cover the most difficult of places to reach in the hilly regions? The answers to the above questions can be addressed by a framework proposed by Arvind Kasthuri as five A’s, namely—Awareness or the lack of it, Access or the lack of it, Absence or the lack of it, Affordability or the cost of healthcare and Accountability or the lack of it [1]. This review of literature is intended to find feasible solution to cater to the above problems in Indian context.

In developing countries, constraints include a lack of suitably skilled employees, poor technical assistance, program management and supervision, insufficient medicine and medical supplies, lack of equipment and infrastructure, and limited accessibility to healthcare services [2]. Inadequate financial resources, workforce, limited infrastructure, health information systems are ineffective., disparity in the availability of services, lack of public participation, as well as a lack of openness and responsibility are just a few of the challenges facing low income countries public health services [3]. Furthermore, poor transportation and communication infrastructure, as well as a scarcity of competent specialists, intensify these issues [4]. As a result, such countries require stronger health care delivery techniques, as evidenced by health care reforms during the last decade [5].

In rural places, quality of healthcare is usually expensive and inaccessible to citizens from lower socioeconomic backgrounds. More than half of pregnant women in South Asia do not receive maternity services, and only one-fifth of deliveries are monitored by doctors. Antiretroviral medication coverage rates for Acquired Immunodeficiency Syndrome (AIDS) in third-world countries are limited to a meagre 5% [6]. In India, over 65% of rural Indians lack access to crucial medicines, and 30% of the rural Indian population travels more than 30 km to seek primary health care [7]. According to a study conducted in Bolivia, 23% of the total reported child morbidities throughout the study period were unable to access medical assistance [8]. According to statistics collected from 42 nations with populations of varied socioeconomic situations, women in the wealthiest quintile were 5.2 times more likely than women in the poorest fifth to receive medical assistance for childbirth [9]. Thus, it can be said that, taking health care to the doorsteps of the poor can be critical to reaching underserved areas.

Mobile Medical Units (MMUs) delivering health care services is a method that has the potential to give high-quality, low-cost health care and transform access to health care for developing-country populations. Furthermore, in economically developing nations, Mobile Health Clinics (MHUs) are a viable choice for both urban and rural populations [10]. According to the literature, Mobile Medical Units (MMUs) have played an essential role in providing not only primary health care but also specialized health care services in rural areas as the major method of delivering healthcare in underdeveloped countries [11–13]. Mobile Medical Units (MMUs) have also shown to be quite effective in bringing health services to India's underprivileged and underserved areas [14]. In the African region, a few studies show that mobile units assisted in cancer diagnosis and treatment, such as skin cancers and cervical cancers [11–13]. As a result, Mobile Medical Units (MMUs) are a viable choice in underdeveloped countries where local health care is inadequate. In areas where fixed facilities are either unavailable or insufficient, Mobile Medical Units (MMUs) can provide not just primary healthcare but also specialized treatments [15].

Considering the above evidence, it is essential to understand the position and role of a mobile Primary Health Center (mPHC) in conjunction with conventional Primary Health Center (PHC) which is a static infrastructure with its own limitations and challenges in the context of healthcare services in rural India.

## Methods

The method followed for the literature search was done in the following manner as stated below:

- *Sources of information* Electronic databases were used when performing literature search which include MEDLINE/PubMed, EMBASE, PsycINFO, Cochrane database, Web of Science, Biomed central and Worldcat library. Search was limited to last 25 years.
- *Search terms and delimiting* An extensive search for all peer-reviewed articles was performed using the keyword “primary healthcare center” and “mobile medical unit”. For a more thorough search, Google Scholar was used. The secondary keywords: developing nations, underserved, underdeveloped, low income, review was used to refine and filter the results for relevancy. As a result, studies focused solely on underdeveloped countries. (Some studies include developed countries but only within the context of hard-to-reach populations), within the time span of last 25 years and in English, were searched.
- *Selection criteria employed* A total of 3870 articles were obtained out of which irrelevant articles were excluded after thorough analysis by both the authors. Hence, a total of 77 articles were selected for the review.

## Results and Discussion

The 77 relevant studies were classified, and issues were synthesized to answer to some of the questions related to its feasibility and scope of the idea proposed. The very first question which is necessary to ask to set the foundation of the study is:

### RQ 1: What is the present healthcare scenario in the remote areas of India? What are the factors responsible for the present situation?

Rural India has roughly 69% of the population but only 26% of hospital beds and 33% of all healthcare professionals [16–18]. Due to unequal distribution of healthcare resources, India's rural public health system is inefficient. An insufficient number of health facilities, frequent drug stockouts, and a persistent human resource shortfall, particularly in rural public health institutions, are the key causes of these inefficiencies [19]. There were 24% fewer government health facilities, 21% less medical doctors, 17.3% fewer nurses, and 14.5% fewer pharmacists providing primary healthcare services in urban areas during 2017 [20–23]. The average rate of absenteeism among the available staff is as high as 40%, and people often have to travel more than 6 km to reach Primary Health Centres [24]. The hard realities of rural life, such as steep terrain, infrequent transportation services, illiteracy, and financial constraints, erect further hurdles to basic health service use, driving individuals to seek care from the unorganized, informal, and expensive private healthcare sector [25]. Only 11.5% of rural households accessed primary level outpatient care (same for childbirth) in public health facilities, according to evidence, and rural households' average medical spending is increasing. For example, in rural India, the average outpatient care expense (per person per fortnight) has climbed from US$29.4 in 2004 to US$55 in 2011 [26, 27]. Thus, to enhance access to healthcare services and safeguard rural people from catastrophic expenditures, it is critical to provide high-quality basic healthcare to them.

Since it has been established that the present healthcare scenario in rural India has infinite barriers to provide basic healthcare services. The next important question which needs to address is:

### RQ 2: Is the mobile Primary Health Center (mPHC), a way forward?

Although, Table 1 establishes that the mobile units are effective way of healthcare delivery, one may argue that what about the telemedicine? How can we compare both? Although telemedicine can be argued as cost-effective and more sustainable way of healthcare delivery, but it is not the reality as stated by Mishra et al. [40] in a study conducted on “Observations in a virtual telephone and WhatsApp video-enabled neurology clinic during lockdown in Varanasi, India” where he mentions that even though after every effort to provide the best advise possible after viewing the diagnostic images on the smartphone, one of the main drawbacks of TeleNeurology Consultation (TNCO) was the low visibility of the radiological image snapshots in the majority of patients. In cases with intracerebral haemorrhage, massive infarcts, ICSOL, and herniated disc, the photographs, which were taken by the patients or family members against a natural backdrop, helped to some extent in establishing the diagnosis. It was hard to accurately detect cases of lacunar stroke, meningitis, brain abnormalities, neurodegenerative disorders, or MS with these photos. However, the written reports from the radiologist transmitted via smartphones substantially resolved the problem. Like other research, this one's shortcomings included the inability to conduct some neurophysiological tests and portions of the neurological examination [41]. Additionally, people from lower socioeconomic strata who couldn't afford cell phones may have received less benefit. Despite proper training, there is variation in the patients' capacity to use their smartphones. This may prevent a clinician from making a timely evaluation [42]. Without suitable caregivers, patients with cognitive dysfunction and visual or auditory impairments may not receive useful consultation guidance. The answers to the patients' and their families' questions, which could have been provided quickly in person, took a lot of time to type in WhatsApp or simple text messages. The lack of proper electronic medical record systems, the need for a telepresenter, and the lack of adequate telemedicine infrastructure at the peripheral level, which is in fact a necessary component of more effective telecommunication, were the other factors in our effective telecommunication setup [43]. Like other studies, a large gap between the demand for and accessibility of neurological treatment in rural areas was also noted. In-person consultations typically do not have these physical, social, linguistic, and financial limitations [44, 45].

**Table 1:** Relevant literatures showing evidence of a mobile PHC, the way forward (Authors own)

Name of the author(s)/project	Country of study	Year of study	Key finding(s) of the study
Labiris G et al. [28]	Greece	2003	Mobile Medical Units are being deployed to provide medical services to underserved communities in remote areas
Siemens Sanjeevan Mobile Clinic [29]	India	2011	Siemens Sanjeevan mobile clinic proves to be better access to primary healthcare services through mobile health unit
Oeltmann JE et al. [30]	India	1995	Mobile Medical Units have potential to provide early screening of difficult to reach populations
Project ‘Mobile Clinic’ Africa [31]	Africa	2015	Effective use of camels to carry the on spot deployable clinic served nest for hard-to-reach populations
Roy D et al. [32]	India	2012	Mobile health services catering to the needs of the far-flung populations by providing minimum basic primary healthcare
Jamir L et al. [33]	India	2013	Mobile health clinics are best to provide healthcare services and its access to the marginalised sections of the society
Van Dijk JH et al. [34]	Zambia	2014	Mobile clinics an effective way of HIV treatment in rural areas
Morrison C [35]	India	1996	Mobile Health Units are efficient ways to execute national health programmes that cover a wide range of services
Mabuto T et al. [36]	South Africa	2014	Mobile Medical Units are efficient in outreach to remote populations for services like HIV counselling and testing
Wangdi K et al. [37]	Asia Pacific	2021	Mobile clinics are best ways for malaria elimination for hard-to-reach populations
Abolfotouh MA et al. [38]	Saudi Arabia	2014	Mobile units will increase in blood donation due to at the doorstep call
Lange M [39]	Kolkata, India	2021	Mobile clinics used for underserved slum population in Kolkata, India
Key issue synthesized	The above studies shows that the mobile PHC would be an effective way of removing barriers against providing services to the remotest of the population as an auxiliary system to conventional static PHC		
Research Gap Identified	The literature only proves the feasibility of the idea, but it lacks to inform the detailed advantages, disadvantages, and limitations to the mobile PHC		

Another study done by Kesavadev et al. [46] on “Telemedicine for diabetes care: An Indian perspective—feasibility and efficacy” discusses the limitation of telemedicine in rural India as patients become agitated by poor communication, slow question responses, a doctor who isn't available to answer the phone, etc. To guarantee quality and expertise in responding to the patient's questions and communicating with them, the interdisciplinary team must undergo rigorous and ongoing training and monitoring. Patients might not be willing to pay more for the teleconsultations despite the numerous advantages. Since telemedicine is built on a patient-centered approach, it is necessary to look at other funding sources in the absence of a clear payment mechanism. A workable option may be to require patients to purchase all their drugs from the hospital pharmacy, which would benefit both the institution by helping to partially fund the telemedicine program and the patient by guaranteeing product quality and sustaining multi-drug adherence [47]. During a telemedicine session, even the smallest communication fault could have catastrophic effects. The telemedicine staff should perform repeated checks on the currently prescribed medications and their dosage before replying to patient inquiries. In India, patients are accustomed to physically visiting the hospital and just receiving medical advice regarding their treatment. The professionally educated interdisciplinary team may need some time to win over the patients' trust and persuade them of the doctor's active role in analyzing their data and changing prescription dosages. Programs for individual and group patient education should cover the advantages and long- and short-term financial viability of telemedicine in the treatment of diabetes. Thus, we can conclude that telemedicine still must overcome many barriers for better healthcare delivery and acceptance in rural India.

Therefore, it has been established from Table 1 that a mobile PHC is a more effective and feasible way forward for providing key healthcare services, it is now the time to address the issue of its implementation on ground.

### RQ 3: What are the factors which should be considered to implement the mobile PHC?

From Table 2, it has been known that a mobile PHC is an alternate solution but not a replacement of the conventional PHC. The mobile PHC cannot function independently, thus it needs a static infrastructure such as facility for accommodation and related services for the mobile staff, nurses, and doctors. The next exploration can be in current services and roles of mobile units:

**Table 2:** Relevant literatures showing evidence on implementation of the mobile PHC (Authors own)

Name of the author(s)/project	Country of study	Year of study	Key finding(s) of the study
Rao SP et al. [48]	India	1999	Assessing the target region health needs and requirements isa prerequisite condition for initiating such services
Ruggiero CP et al. [49]	Costa Rica	1995	Collaboration between screening and surgical camps will help to increase coverage and usage
Patro B et al. [50]	India	2008	Complicated or high order cases when referred to specialized Community Health Center (CHC) or district hospitals enhances the effectiveness of the mobile unit
Kar CG et al. [51]	India	2007	a) Provision of office, staff, and storage space for the medical health units at the deployment site b) Adequate number of MBBS doctors must be posted c) Training for medical and paramedical workers should be organised on a regular basis d) It is necessary to create awareness through school visits, immunization camps, antenatal check-ups, health camps, family welfare camps, etc
Morrison C [52]	India	1996	Mobile Health Units had 1 medical officer, 1 field coordinator, 4 field workers. 1 auxiliary nurse midwife, and 1 driver
Lindgren TG et al. [53]	Malawi, India	2011	a) According to the user requirements assessment, a schedule of required services should be given b) Community based publicity campaigns encourages the residents to turn to the mobile facility more and thus enhances the utilization c) The unit should be prepared for re-prioritizing in times of needs and demands of the population d) Understanding the factors and constraints for utilization in some context and under-utilization in the other for better planning before deployment in resource constrained emergent countries
Key issue synthesized	The above studies clearly state some of the general guidelines for implementation of mobile PHC. Though it is not a detailed guidelines but enough to understand the way forward		
Research Gap Identified	There is a lack of detailed guidelines on how to implement the mobile PHC on ground level		

### RQ 4: What are the different services which a mobile PHC can cater in India? What are the factors that will govern the type of services?

Table 3 indicates some services which can be catered by the mobile PHC, but there are other details in terms of its operation based on different geographical locations, which is still unknown. Since there are different geographical factors, and each has its own limitations in terms of its accessibility like deploying a mobile PHC on a hilly region is a challenge and other remote locations in rural regions where the road conditions are pathetic in Indian context.

**Table 3:** Relevant literatures showing evidence of services which can be catered by a mobile PHC (Authors own)

Name of the author(s)/project	Country of study	Year of study	Key finding(s) of the study
MoFHW-NHM guidelines [54]	India	2015	Antiretroviral therapy (ART) is increasingly being offered in fixed clinics, and mobile clinics play a key part
Alam MF et al. [55]	Bangladesh	2013	Mobile Medical Units used by vision -impaired patients in remotest of the locations in Bangladesh
Rizk HI et al. [56]	Egypt	2021	Family planning campaigns in mobile clinics helped in increasing contraceptive coverage rate in Egypt
Amimo F et al. [57]	Africa	2021	Mobile clinics are used as community-based strategies to provide equitable access to vaccines in Africa
Kojima N et al. [58]	Mysore, India	2017	Mobile medical clinics are effective tools for promoting, educating, and treating people's health
Al-Oraibi A et al. [59]	Jordan	2021	Mobile clinics are best for outreach activities among refugees
Tahir ARM et al. [60]	Rohingya	2021	Detecting and treating infectious diseases are best tackled by mobile clinics among Rohingya paediatric community
Kamili I et al. [61]	Rwandan	2021	Treatment of hepatitis C in mobile clinic in Rwandan district
Khatiwada AP et al. [62]	Nepal	2021	Immunisation services are provided through mobile clinics among children in Nepal
Wangdi K et al. [37]	Asia pacific	2020	Mobile clinics ensure that malaria patients in border or forested areas have access to effective therapies
Kumar P et al. [63]	Bihar and UP, India	2021	Mobile clinics can be utilized to disseminate relevant information to teenagers and to evaluate asymptomatic adolescents for gynaecological morbidity
Smith PJ et al. [64]	Cape Town, South Africa	2021	Mobile clinics are effective in reaching the underserved Africans for chronic disease screening, HIV counselling and testing
Baker DE et al. [65]	Uganda	2021	Mobile clinics helps in improving diagnosis capabilities through point of care ultrasound in patients of rural Uganda
Msokwa R [66]	Malawi	2021	Mobile clinics can help to screen diseases, provision of primary healthcare, and manage conditions among elderly
Yang Y et al. [67]	Developing nations	2019	Mobile clinics have proven to be an effective strategy to reach out to isolated people that do not have easy access to clinics and hospitals for new-born vaccines
Saka B et al. [68]	Togo, Africa	2021	Mobile clinics used for skin care in remote populations of Togo
Key issue synthesized	The above studies shows that there is a wide range of services which a mobile PHC can cater to remote populations depending upon the intended need		
Research gap identified	Though literature suggests that there can be many possibilities of services which can be provided by a mobile PHC, but it does not clearly state the factors that will govern the type of services. Is the factor being only the intended need or are there other limitations to it?		

Thus, it is imperative to ask the most important questions which are as follows:

- Q5: What kind of design solution will work in different geographical regions of India? Is the design solution same for hilly region, plain region and during a disaster, etc.? What are the factors which will govern the design of the mobile PHC?
- Q6: What are the constraints in the adoption and operationalization of a mobile PHC? What are the feasible methods to overcome such limitations?
- Q7: What will be the method of operation of a mobile PHC during different scenarios such as landslides, floods, earthquakes, etc.? How the mobile PHC will be deployed in such situations?

It is found that there are no literatures which addresses the above-mentioned issues. Thus, these are important research gaps in the literature pertinent to mobile Primary Health Centers (mPHCs).

### State of the art

The 19 state-of-the-art that the author’s found relevant were analysed. Issues were synthesized to answer some of the concerns about its operationalization, services provided, advantages and disadvantages of the system installed, resulting in a better understanding of areas where future interventions of this type could be improved.

The following are the key takeaways for future interventions from the state of the art (mentioned in Table 4):

- The system designed is based on the constraints and limitations of the context. Thus, it is important to derive design solutions by studying the context in enquiry.
- The system designed should have modularity so that it can be extended as per needs.
- The system designed should have portability so that it can relocated as per needs.
- The system should be self-sustainable in terms of a clean sterile environment, water storage provision, and electricity backup facility.
- The focus should be on the structural system devised for easy deployment.
- The size and weight of the system designed should be according to the context.

**Table 4:** State of the art (Authors Own)

Project Name/Country/year	Method of operation and services provided	Key findings of the study
Mobile Medical vans—Nargis Dutt Foundation [69]	Free medical camps being held at Kadeshwari (Bandra West) and Golibar (Santacruz East) in Mumbai for those in need. The van is stationed at the intended place. The retractable roof system installed on top of the van is opened, tables and chairs were parked below the roof which acts as an OPD	Use of retractable roof to conduct OPD
Smile on wheels—Smile Foundation [70]	Mobile medical vans providing people in need with the necessary doorstep healthcare services through ‘Smile on Wheels’ initiative. An independent retractable roof is installed near the van for OPD services	Use of collapsible system to conduct OPD
Camel Mobile Clinic, Africa, 2011 [71]	Nairobi's thorny scrubland and semi-arid bush were traversed by camel. The convoy pitches the tent near a huge manyatta (a traditional homestead erected by a family or a clan) for a minimum of two or more days stay of the medical team after arriving in the destination. Serves 30–80 people in one group before moving on to the next. Screening services, blood and sugar tests, and a pharmacy for medication dispensing are also available	Effective use of camels to carry the on spot deployable clinic served best for hard-to-reach populations
Siemens Sanjeevan Mobile Clinic, India, 2015 [29]	It is a retrofit on a minibus that travels to the Kalyan-Dombivali municipal region in Mumbai city's informal communities. When they get at their destination, they board the bus for the appropriate diagnostic based on their on-the-spot registration. The bulk of migrant labourers, such as daily wage employees, construction workers, sellers, and others, are served. Basic diagnostics such as x-rays and ECGs are available, as well as a pathology lab for blood, sugar, and other basic tests	Siemens Sanjeevan mobile clinic proves to be better access to primary healthcare services through the mobile health unit
Mobile Eye Surgical Unit, India, 2011 [72]	Cataract surgery can be performed in remote areas using a self-contained, sterile surgical unit. Two vans are set up, one for the preparatory, which includes a prep room and a changing room, and the other for the surgical vehicle, which includes an OT, a scrub, and a sterilising room. Serves cataract surgery in Tamil Nadu's distant areas	Mobile medical units have the potential to provide early screening of difficult to reach populations
Project 'Mobile Clinic' Africa, 2012 [31]	A mobile clinic vehicle for maternity and child health care, custom-built on a mid-sized truck frame of 10 m by 2.5 m. The vehicle's most remarkable feature is the inclusion in the design of the entry gate, which is significantly closer to the ground to make it easier for youngsters and pregnant women to embark. Immunization and antibiotics are given to children under the age of five to avoid illnesses such as acute respiratory infections, diarrhoea, malaria, and other ailments. Pre-natal and post-natal care should be provided to the mothers	Custom made medical units deployed to provide medical services to under-served remote populations
Uganda Village Ambulance, Africa, 2013 [73]	A three-wheeled motorcycle on which medical supplies and information are delivered to patients. It makes it easier to access the most remote areas, where health professionals traditionally had to walk with patients on a stretcher. Provides medicine and health information to the residents of the village. Used to assist in the event of an emergency, such as a baby delivery or a car accident	An innovative detachable design of a tricycle to commute to the hardest of the locations in villages of Uganda
Mohalla Clinic, India, 2021 [74]	A clinic built in a shipping container that is prefabricated off-site and installed in a week. Provides the necessary screening and diagnosis services to the marginalised at a low cost. There is a consultation room, as well as test samples, waiting, and a restroom	Module portability from one place to the other provides high flexibility
Mobile Medical Vans—Ziqitza Healthcare Limited[75]	Mobile medical vans cater for medical emergency response. In both the first and second phases of the epidemic, Ziqitza was instrumental in assisting people, and it is now gearing up for many such events in the future to assist individuals in getting to hospitals and ensuring that they are returned home	Use of a Mobile Medical Van service to transfer patient at times of need
Relief on the Move – HelpAge’s Mobil-health service [76]	HelpAge's Mobile Healthcare project strives to provide sustainable healthcare solutions to poor elders and their communities through its Mobile Healthcare Units (MHUs). Each MHU has a doctor, pharmacist, and social worker on staff. These mobile health units (MHUs) move deep into urban slums and villages, providing healthcare to the poorest of the poor. It benefits most of the elderly because it keeps them out of huge lines at hospitals that are also far away from their homes, and they receive free medication monthly. Their one-of-a-kind patient card records their therapy and allows them to monitor their progress	MMU with an addition patient card records make it a more efficient system in providing primary healthcare service
Varisthajana Swasthya Sewa Abhiyan [77]	Mobile Medicare Units are being developed by ONGC in conjunction with Help Age India to offer healthcare to the elderly's doorsteps. To provide vital medical services to the elderly at their homes, such as medical consultations, medicine distribution, basic diagnostic tests, special health camps, and palliative care	MMU to treat elderly at homes
The Floating Homoeopathy Dispensary [78]	The major goal is to make high-quality health care more accessible to people in rural regions, particularly disadvantaged women, and children. Children and the elderly living in the backwaters have benefited from the effort of transforming a vessel into a hospital, as it is difficult for them to travel to hospitals on the mainland. Two Homoeopathic Medical Officers, a Pharmacist, an Attendant, and enough medicines will be available at this dispensary	MMU to treat women and children
Deendayal Mobile Hospital Scheme [79]	The Deendayal Mobile Hospital Scheme was established in June 2006 with the goal of bringing high-quality health care to the state's rural districts. A mobile van is outfitted with a doctor, staff, necessary appliances, and medicines as part of the programme. Free medical treatment is provided to patients in tribal-dominated areas and Haat-Bazaars by this vehicle. Tribals who live in remote rural and forest locations are rarely able to visit a hospital. They also avoid going because there aren't enough facilities. This plan has provided people with high-quality healthcare and therapy in their villages and haat bazaars, which is nothing short of a blessing	MMU for tribal people
Chief minister urban slum health scheme [80]	To give those living in slum areas with high-quality free health consultations, check-ups, treatment, and medicines. People living in slum areas must be informed about family planning methods by personal dialogue and other ways, and family planning resources must be made available. Women living in slum areas will be offered free ANC/PNC tests. If significant diseases are discovered during a health check-up, residents of slum regions must be directed to district hospitals/higher health facilities. To deliver health-related information in the slum region through health education, as well as information on prevention strategies	MMU for the urban slum dwellers
Tata Power’s Integrated Community Health Care [81]	Providing solutions for better health care. Mobile medical units service to provide healthcare facilities at the doorstep	MMU at each household
Dhanwantri [82]	The main goal of the project is to bring awareness, diagnostic, and therapeutic services to the rural community via Mobile Medical Units	MMU acting as a mobile awareness camp
Sachal Swasthya Seva – Indian Oil Corporation [83]	The goal for Mobile Medical Units under this initiative to give free medical check-ups and medicines to locals	MMU serving the deepest of the community
Jankidevi Bajaj Gram Vikas Sanstha [84]	Aim to treat Communicable and Non communicable diseases with special focus on Mental Health in Mobile medical units	MMU used to treat for mental health
GMRVF Mobile medical units—Varalakshmi Foundation [8]	GMRVF operates Mobile Medical Units that provide care to the elderly and disadvantaged who might otherwise be unable to receive it. The vans are stocked with basic medical supplies and medications, as well as a doctor, pharmacist, and social worker on board. MMUs visit communities in GMRVF sites on a regular basis. At each place, there exist nodal points. Each nodal point is visited once a week by the vehicle. These MMUs are frequently organized in collaboration with specialized non-governmental organizations. MMUs address common ailments such as osteoarthritis, hypertension, chronic obstructive pulmonary disease, and dyspepsia on a regular basis. If necessary, referral services are also available	MMU acting as a referral service

Thus, the above case studies paved a path for future interventions in making the Primary Health Centres (PHCs) mobile. It is thus needed on the part of the designers, engineers, health professionals, and policymakers to learn from the state of the art, the advantages, disadvantages, shortcomings and to implement the same for the much-needed mobile PHCs to make the healthcare services reach the doorstep of the remotest of the populations.

## Limitations and Future Scope

The study has limitations in terms of adequate search of literatures to answer the later posed questions. Though the above state of the art explains the current systems only in the context of services provided and overall design of medical units but fails to provide answers to the later posed Q5, Q6 and Q7. Answers to these questions needs a separate search strategy and analysis which can be done in future research.

## Conclusion

We can conclude that there are several evident lacunae in the literature regarding the alternative way of providing the basic key primary healthcare services for hard-to-reach populations. According to this study, Mobile Medical Units (MMUs) provide several advantages, including the ability to be conveniently transportable while providing better patient care coverage, as well as assisting in the delivery of high-quality primary and specialised health care in developing nations. It's also important to remember that the efforts are still in their infancy and are limited by a range of organisational, financial, human, and operational obstacles. The concept of a mobile Primary Health Center (PHC) should be carefully considered before being implemented. For a successful deployment, preliminary investigations to identify the needs of the population and challenges of the context are required. Effective public relations campaigns, as well as the ability to refer to higher authority centres via mobile services, improve community mobilization and, as a result, its usage. Furthermore, assessment of patient satisfaction at regular intervals by asking feedback is an important part of the implementation process. It's critical to understand why something isn't being used to improve and create a suitable foundation for policy analysis. The long-term feasibility of this type of health-care delivery model will necessitate more study and in-depth investigations in the future, employing a variety of research methods. Finally, it can be said that indeed Primary Health Centers (PHCs) can be made mobile for a more efficient delivery of healthcare services for remotest population. Thus, we propose an innovative concept in the mobile health unit infrastructure to provide key healthcare to the underserved population.

Healthcare on Wheels—Mobilizing healthcare to the doorstep of the remote populations—A lesson learned from the Covid-19 pandemic. The Indian healthcare system urgently requires a new healthcare delivery model to improve healthcare accessibility and health outcomes for the marginalized. People living in remote areas are disproportionately affected by the inaccessibility and underutilization of Primary Health Centers (PHCs). The goal is to design, develop and deploy a cost-effective collapsible mobile Primary Health Center (mPHC) unit in low resource settings. A traditional PHC activities will be decentralized into several modules for ease of deployment and reachability to remote locations. The proposed concept of a collapsible system for a mobile Primary Health Center (mPHC) is intended to be carried in bags to remote regions, deployed in less than 60 min, run OPD for 4–6 h, collapse, and return to the base camp. It is based on the concept of patient-centered care, with healthcare delivered to the underprivileged's doorstep. Most of the system's elements must be collapsible by design for it to be collapsible. As a result, it is critical to focus on the design of sub-systems to be as collapsible as possible so that take up the least amount of space for easy transport.

## Data Availability

Not applicable.
